# Dental Metallurgy

**Published:** 1888-04

**Authors:** 


### BIBLIOGRAPHICAL.

### Dental Metallurgy.

A MANUAL FOR THE USE OF DENTAL STUDENTS.

BY CHAS. J. ESSIG, M.D., D.D.S.

Professor of Mechanical Dentistry and Metallurgy in the Dental Department of the University of Pensylvania.

SECOND EDITION.—REVISED.

This little work, just published by the S. S. White Dental Manufacturing Co., is one the general character of which is well known to the profession. It is a most excellent and reliable work upon the subject of Metallurgy, and better adapted to the wants of the dentist for a ready reference than any work with which we are familiar. This edition has been carefully revised, and while it has not been greatly enlarged, the more recent improvements in the working of metals, the formation of alloys and amalgams used in dentistry, have been embodied. All extraneous matter has been carefully put aside, and only that presented which is necessary to convey the facts and principles pertaining to the subject. Probably no one in the country is better, if as well, qualified for the preparation of such a work as Professor Essig. He has long been engaged in teaching in this line, and his knowledge of principles and practical details are a sufficient guarantee as to the character of the work here presented. It is a work that has already taken its place in the list of text books in our colleges, and it should be in the library of every dentist.

